# Biodiversity of *Ligilactobacillus salivarius* Strains from Poultry and Domestic Pigeons

**DOI:** 10.3390/ani11040972

**Published:** 2021-03-31

**Authors:** Marta Dec, Dagmara Stępień-Pyśniak, Andrzej Puchalski, Tomasz Hauschild, Dorota Pietras-Ożga, Szymon Ignaciuk, Renata Urban-Chmiel

**Affiliations:** 1 Sub-Department of Veterinary Prevention and Avian Diseases, University of Life Sciences in Lublin, 20-033 Lublin, Poland; marta.dec@up.lublin.pl (M.D.); dagmara.stepien@up.lublin.pl (D.S.-P.); andrzej.puchalski@up.lublin.pl (A.P.); 2 Department of Microbiology and Biotechnology, Faculty of Biology, University of Bialystok, 15-245 Białystok, Poland; thausch@uwb.edu.pl; 3 Department of Epizootiology and Clinic of Infectious Diseases, Faculty of Veterinary Medicine, University of Life Sciences in Lublin, 20-612 Lublin, Poland; dorota.ozga@up.lublin.pl; 4 Sub-Department of Mathematics, Department of Applied Mathematics and Computer Science, Faculty of Production Engineering, University of Life Sciences in Lublin, 20-612 Lublin, Poland; szymon.ignaciuk@up.lublin.pl

**Keywords:** *Ligilactobacillus salivarius*, 16S-23S rDNA, morphology, biofilm, carbohydrate fermentation, bacteriocin, phylogenetic, hydrophobicity, bile resistance

## Abstract

**Simple Summary:**

*Ligilactobcillus salivarius* is a Gram-positive bacterium that commonly colonizes the mucous membranes of the digestive tracts of humans and animals, including birds. It belongs to the group of lactic acid bacteria which, by producing lactic acid, acidify the intestinal environment and limit the development of undesirable intestinal microflora. In addition, *L. salivarius* can produce other antimicrobial substances, such as bacteriocins and hydrogen peroxide. Due to limiting the development of unfavourable microflora and other health-promoting effects, *L. salivarius* bacteria are considered as potential probiotics that may increase animal health, and thus animal production indicators. In this work, we undertook research on the characteristics of *L. salivarius* strains from chickens, geese, turkeys and domestic pigeons. We showed great variation in phenotypic and genotypic traits between strains and the evolutionary adaptation of *L. salivarius* strains to the colonization of a specific host. The results of the study contribute to knowledge of the characteristics of the species *L. salivarius* and may be useful in the selection of probiotic strains.

**Abstract:**

*Ligilactobacillus salivarius* is an important member of the human and animal gut microbiota, and selected strains are promising probiotics, but knowledge of the characteristics of avian isolates is still limited. In this study, we examined selected phenotypic and genotypic traits of 33 *L. salivarius* strains from geese, chickens, turkeys and pigeons. The strains varied in terms of cell size, colony morphology, broth growth characteristics, biofilm formation, tolerance to bile, hydrophobicity and phenotypic and genotypic antibiotic resistance profiles. Large variation among strains was noted for the utilization of sorbitol, salicin, trehalose, rhamnose, inulin and N-acetyl-D-glucosamine. The presence of genes related to sugar metabolism, i.e., *mipB*, *tktA*, *rhaB* and LSL_1894, was not always correlated with the biochemical phenotypic profile. Correlations were recorded between the host and utilization of certain sugars as well as tolerance to bile. The *repA*-type megaplasmid and genes coding for Abp118 bacteriocin were detected in 94% and 51.5% of *L. salivarius* strains, respectively. Phylogeny based on *groEL* gene sequences was partly correlated with the origin of the strains and revealed an evolutionary distance between *L. salivarius* strains from humans and birds. The results of the study contribute to knowledge of the characteristics of the species *L. salivarius*. Intraspecies variations of *L. salivarius* strains may affect their ability to colonize specific niches and utilize nutrients and reveal potential strain-dependent effects on host health.

## 1. Introduction

The species *Ligilactobacillus salivarius* was originally described in 1953 by Rogosa et al. [1] as *Lactobacillus salivarius*. This species name was used for nearly 70 years, but in accordance with the new taxonomic classification of *Lactobacillaceae* and *Leuconostocaceae*, the genus *Lactobacillus*, comprising about 260 species until March 2020, was divided into 25 genera, and the name *L. salivarius* was changed to *Ligilactobacillus salivarius* [2].

*L. salivarius* bacteria are Gram-positive, non-motile, non-spore-forming, catalase-negative, aerotolerant anaerobic rods commonly isolated from the intestines or faeces of birds and mammals, including geese [3], chickens [4], turkeys [5], pigeons [6], ducks [7], pigs [8] and cattle [9,10]. The presence of *L. salivarius* has also been confirmed in the oral cavity [11] and vagina of humans [12], in human breast milk [13] and in the gut of honeybees [14], as well as in grape wine [15], meat [16] and St. Ivel cheese [17].

The species *L. salivarius* mainly comprises homofermentative strains that ferment hexoses to lactic acid in the Embden–Meyerhof–Parnas (EMP) pathway, but heterofermentative strains of *L. salivarius* (e.g., UCC118) are also known. The latter have been shown to be able to degrade ribose via an inducible phosphoketolase, an enzyme of the pentose phosphate pathway, and to produce lactic acid, acetic acid and ethanol from hexoses. Some strains have D-lactate dehydrogenase, meaning that in addition to L(+) lactic acid, they can produce the D(−)-isomer [18,19]. The average genome size of *L. salivarius* strains is 2.14 ± 0.14 Mbp, with 2062.74 ± 134.26 genes and a GC content of 32.84 ± 0.12% [8]. A characteristic feature of *L. salivarius* is the presence of 100–380 kbp *repA*-type megaplasmids and small plasmids in the cells. Extra linear and circular megaplasmids are less common [20]. Studies conducted thus far have shown significant genotypic differentiation among *L. salivarius* strains, in both chromosomal and plasmid sequences. The greatest variability has been noted in genes encoding glycosyl hydrolases, bacteriocins and proteases, as well as genes responsible for exopolysaccharide synthesis [20].

*L. salivarius* has the Qualified Presumption of Safety (QPS) status granted by the European Food Safety Authority (EFSA) to biological agents considered safe for use [21], and several authors have demonstrated the antimicrobial activity of avian *L. salivarius* strains against bacterial pathogens, including *Salmonella enterica*, *Clostridium perfringens*, *Staphylococcus aureus*, *Pasteurella multocida*, *Riemerella anatipestifer* and *Campylobacter* spp. The competitive exclusion of unfavorable microflora by *L. salivarius* strains depends on the production of lactic acid, hydrogen peroxide and bacteriocins, as well as the ability of the *L. salivarius* strains to permanently colonize the intestine [4,22,23,24,25]. A wide range of bacteriocins produced by *L. salivarius* strains include class II salivaricins, i.e., salivaricins P, T, L, CRL 1328, LS1, LS2 and Abp118, which have been found in human and porcine strains and salivaricins SMXD5, FK22, OR7 and L-1077, which can be secreted by *L. salivarius* strains from poultry [22]. Despite the documented antimicrobial activity of avian *L. salivarius* strains, they are not often used as feed additives for poultry, and the best described probiotic strain to date is UCC118 from resected human terminal ileum. It shows tolerance to gastric acid, resistance to bile, enhanced adhesion to the human intestinal epithelial cells, antimicrobial and anti-inflammatory activity, and its genome is used as a reference point in various genetic analyses [26].

The aim of this study was to determine selected phenotypic and genotypic traits of *L. salivarius* strains from chickens, geese, turkeys and domestic pigeons, including growth characteristics, cell size, the ability to utilize various carbon sources, biofilm formation, bile tolerance, hydrophobicity and antibiotic susceptibility, as well as the presence of resistance genes, *repA*-type megaplasmids and genes involved in sugar metabolism. The determination of the intraspecies diversity of *L. salivarius* strains will contribute to a better understanding of the biological compatibility of these bacteria with the host, their biological activity and may be helpful in the selection of probiotic strains.

## 2. Materials and Methods

### 2.1. Bacterial Strains and Culture Conditions

A total of 33 wild-type strains of *Lactobacillus salivarius* were used in the study. They were isolated from the fresh faeces of Green-legged Partridge hens (3 strains, Ch4b-Ch9b), broilers (7 strains, Ch10a-Ch50d), turkeys (9 strains, T2-T31a), domestic geese (7 strains, G2K-G50b) and domestic pigeons (7 strains, P2a-P23a) from lofts and farms located in Poland. Faecal samples were obtained from asymptomatic adult birds and were collected between 2013 and 2017 by veterinarians who supervised individual poultry flocks. Chickens were reared in a standard litter system, geese and turkeys in a semi-open system and pigeons in two-pen lofts. The diet of the birds did not contain microbiological feed additives and was adequate for the age, species and utility breed of the birds and the season. Bacteria were cultured in Man–Rogosa–Sharpe (MRS) medium supplemented with L-cysteine (0.05% *w*/*v*) at 37 °C, 5% CO_2_, and stored at −80 °C in MRS broth containing ~20% glycerol. Reference strains of *L. salivarius*, i.e., LMG 9476 (formerly *L. salivarius* subsp. *Salicinus*) and LMG 9477 (formerly *L. salivarius* subsp. *Salivarius*), as well as *L. agilis* LMG 9186 and *L. saerimneri* LMG 22875, were purchased from the BCCMTM/LMG Bacteria Collection (Gent, Belgium).

### 2.2. Identification of L. salivarius

*L. salivarius* strains were identified by MALDI-TOF mass spectrometry [27] and by sequence analysis of the *groEL* gene [18]. Additionally, the taxonomic affiliation of some of the isolates tested in this work (Ch8b, Ch10d, Ch24b, Ch50b, G2K, G19a, G24a, G24b, G31a, G39a and G50b) was determined in our previous studies based on the analysis of 16S-23S rDNA regions [3] and/or 16S rDNA [28].

A standard ethanol/formic acid extraction procedure was used to identify the strains in an UltrafleXtreme MALDI-TOF mass spectrometer (Bruker, Germany). Briefly, the 2–3 bacterial colonies were suspended in 300 µL of ultrapure water, and then 900 mL of absolute ethanol (≥99.8%) was added. In this form, the samples were kept at −20 °C up to 1 month. On the day of analysis, the mixture was centrifuged (13,000× *g*, 2.5 min), the supernatant removed and the bacterial pellet was resuspended in 50 µL of 70% formic acid. After vortexing (1 min), 50 µL of ≥99.9% acetonitrile was added and the resulting mixture was vortexed (1 min) and centrifuged (13,000× *g* for 2.5 min). Aliquots of 1 µL of the supernatant of each sample were spotted onto three positions of the 384 MTP AnchorChip TF stainless steel MALDI target plate (Bruker, Germany), overlaid with 1 μL matrix solution (HCCA a-cyano-4-hydroxycinnamic acid, Bruker Germany, dissolved in 50% acetonitrile/0.1% trifluoroacetic acid, 10 mg/mL) and allowed to co-crystalize for 1 min. Calibration was preceded with a bacterial test standard (*E. coli* DH5 alpha, Bruker Daltonics). Microbial mass spectra were analysed using MALDI Biotyper 3.1 software (Bruker, Germany) containing 5989 reference spectra, including 3 strains of *L. salivarius* [27]. The identification score criteria used were those recommended by the manufacturer: score ≥2.300 indicates a reliable species-level identification; 2.00–2.299, probable species; 1.700–1.999, a genus-level identification; and a score <1.700 indicates no reliable identification. The relationship among MSPs obtained from each strain was visualized in a score-oriented dendrogram using the unweighted pair group method with arithmetic mean (UPGMA) implemented in the MALDI Biotyper 3.1 software package.

The *groEL* gene was amplified using primers LSL_1212 groS_F2 (59-AAACCATTAGGAGATCGCGTT) and LSL_1211groL_R2 (59-ATCATACCGCCCATACCTG) [18]. The PCR product of ~1900 bp was sequenced using the Sanger method (Genomed, Warsaw, Poland) and compared to the sequences available in the GenBank database using the National Center for Biotechnology Information (NCBI) Basic Local Alignment Search Tool (BLAST). DNA sequences were deposited in GenBank (Accession Nos. MT862775-MT862808 and MT920907, Table A1 in Appendix A).

### 2.3. Morphology and Growth Features

L. salivarius cultures were Gram-stained, and cell size was assessed using an Olympus DP72 microscope and Cell˄F 3.1 software. The morphology of the colonies and the ability of L. salivarius strains to grow at 45 °C in an atmosphere containing 5% CO_2_ and at 37 °C under aerobic conditions were also assessed (the growth intensity was visually compared with respect to cultivation under standard conditions, i.e., 37 °C, 5% CO_2_). Suspension formation during the growth of the strains on MRS broth was visually assessed according to the following scale: − growth in the form of sediment; + low suspension; ++ high suspension. To assess the ability of L. salivarius bacteria to self-aggregate, tubes with 24-h MRS broth cultures were vortexed vigorously for 90 s, and the presence of bacterial aggregates in the broth was visually assessed (−, +, ++).

### 2.4. Fermentation Assay

Twenty-four carbohydrates, including pentoses (D/L-arabinose, ribose and xylose), hexoses (glucose, galactose, fructose and mannose), 6-deoxyhexoses (rhamnose and fucose), disaccharides (cellobiose, maltose, lactose, melibiose, saccharose and trehalose), trisaccharides (D-melezitose and D-raffinose), polysaccharides (inulin), sugar alcohols (mannitol, sorbitol and xylitol), alcoholic β-glucoside (salicin), N-acetylglucosamine (GlcNAc, an amide derivative of the monosaccharide glucose) and amygdalin (cyanogenic glycoside), were used in the experiment. All sugars were purchased in powdered form from Sigma-Aldrich (Poznań, Poland). The fermentation assay was performed according to the protocol developed by Hedberd et al. [29]. Two-percent aqueous carbohydrate solutions were made, filtered using 0.45 µm syringe filters and stored for no longer than 2 weeks at 4 °C. Freshly grown bacterial cultures were suspended in 0.85% NaCl so that the optical density was 1.0 on the McFarland scale. A 50 µL volume of modified MRS broth (pH 6.7) without carbohydrates was mixed with 50 µL each of 24 carbohydrates, 5 µL of sterile filtered 2% solution of bromocresol purple and 5 µL of bacterial suspension in the wells of microtiter plates. In the negative control, 0.85% NaCl was added instead of bacteria. Plates were incubated at 37 °C, 5% CO_2_, and examined for colour changes after 24, 48 and 72 h. Purple (pH > 6.8) was considered a negative result (−), yellow (pH < 5.2) indicated a positive reaction (+) and an intermediate colour (pH between 5.2 and 6.8) was considered poor fermentation (+/−). The experiment was performed in two independent repetitions.

### 2.5. Detection of repE, Abp118 Bacteriocin Genes and Genes Associated with Sugar Metabolism

The *repE* gene, which is a molecular marker of the *repA*-type megaplasmid characteristic of *L. salivarius*; the *abp118β + abp118α* gene coding for Abp118 bacteriocin (beta + alpha peptide); and the genes involved in the metabolism of rhamnose, sorbitol and pentoses, i.e., *rhaB* (coding for rhamnulokinase), LSL-1894 (sorbitol-6-phosphate 2-dehydrogenase), *mipB* (transaldolase) and *tktA* (transketolase), were detected using primers previously developed by Li et al. [30] (Table 1).

Due to the heterogeneity of the size of the *abp118β* + *abo118α* amplicons, those selected were sequenced using the Sanger method and compared to reference sequences available in the GenBank database using the BLAST algorithm.

### 2.6. Phylogenetic Analysis

The *groEL* gene of the 33 wild-type and 2 reference *L. salivarius* strains were amplified and sequenced as described above (Section 2.2). The amino acid sequences were predicted using the NCBI translate tool ORF finder [31]. With MEGA X software, the *groEL* sequences were aligned using the ClustalW program, and the maximum likelihood method was used to create phylogenetic trees with a bootstrap support value of 500. The analysis included 30 additional *L. salivarius* strains, mainly of human origin [18], whose *groEL* sequences were retrieved from the NCBI genome database [32].

### 2.7. Bile Tolerance Test

The tolerance of *L. salivarius* isolates to bile salts was determined in a microplate assay. MRS medium (200 µL) containing 1% or 2% ox gall (BTL, Łódź, Poland) was inoculated with 0.5 µL of fresh broth cultures of lactobacilli. Following 24 and 48 h incubation at 37 °C, 5% CO_2_, the optical density of the bacterial cultures was measured at 620 nm. Positive controls were bacterial cultures grown without ox gall. The growth of each strain was expressed as a percentage of the OD620 value of the control samples [4]. To check the bacterial viability in the presence of bile, after 24 h of incubation, 10 µL was pipetted from each well and added to fresh MRS broth. Bacterial growth was assessed after 48 h.

### 2.8. Biofilm Formation

MRS broth was dispensed in 200 mL volumes into the wells of Nunc MaxiSorpTM 96-well flat-bottom plates (Biokom, Janki, Poland) and inoculated with 1 µL fresh bacterial culture. Following 48 h incubation at 37 °C, 5% CO_2_, the wells were emptied and washed 3 times with 0.85% NaCl. Adherent cells were stained with crystal violet (1% *w*/*v*, 50 µL) for 15 min. Unbound dye was washed off with water, and cell-bound dye was dissolved in 20% acetone in ethanol for 10 min; the absorbance (A570) was measured using a Microplate Reader 680 (Bio-Rad, Warszawa, Poland). Isolates were classified as follows: no biofilm producer (−), weak biofilm producer (+), moderate biofilm producer (++) and strong biofilm producer (+++), based on the absorbance value [23].

### 2.9. Measurement of Bacterial Hydrophobicity

The hydrophobicity of the bacteria was determined on the basis of microbial adhesion to xylene, as described previously [4]. Strains with hydrophobicity equal to or greater than 50% were considered hydrophobic.

### 2.10. Phenotypic and Genotypic Profiles of Antimicrobial Resistance

The antimicrobial susceptibility of L. salivarius strains was determined by the broth microdilution method, as described previously [6]. The following antimicrobial agents were included in the study: ampicillin, tetracycline, streptomycin, gentamicin, kanamycin, erythromycin, lincomycin, chloramphenicol and enrofloxacin. The categorization of strains into susceptible or resistant was based on the MIC cut offs recommended by EFSA [33]. For enrofloxacin and lincomycin that are not included in the EFSA guidelines, strains with MIC values above 32 µg/mL were considered resistant [6].

In order to determine the genotypic profiles of antimicrobial resistance in the tested strains, a number of resistance genes, i.e., *tetK, tetL, tetM, tetO, ermA, ermB, ermC, mefA/E, lnuA, aph(3′)-IIIa, aac(6′)-Ie-aph(2″)-Ia, aph(2″)-Ib, aph(2″)-Ic, aph(2″)-Id, ant(4′)-Ia, ant(6)-Ia, cat, lsaE* and the Tn916 integrase gene *int-Tn*, were detected using the primers and the methodology described in our previous papers [5,6].

### 2.11. Statistical Analysis

Yates’s chi-squared test was used to compare the frequency of occurrence of strains utilizing individual carbohydrates among the pools of strains from pigeons, chickens, turkeys and geese. The same test was also used to determine the relationship between the host and the ability of the strains to grow in bile-supplemented medium. The power of the relationships was assessed with contingency coefficients (C), which ranged from 0.000 to 0.707 for the tests. The level of significance was set as *p* < 0.05. The statistical analysis was performed using Microsoft Excel 2019.

## 3. Results

### 3.1. Identification of L. salivarius Strains

**Figure 1:**
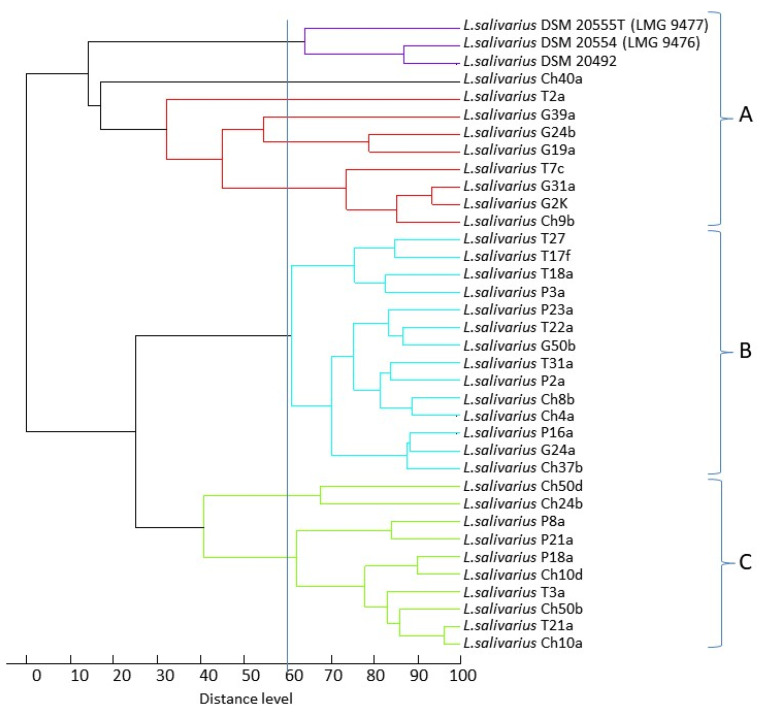
Dendrogram derived from UPGMA cluster analysis of MALDI-TOF mass spectra obtained from the 33 wild-type *L. salivarius* strains. The relative distance between isolates is displayed as arbitrary units. One hundred indicates complete similarity and 0 indicates maximum dissimilarity. The vertical line corresponds to the level of similarity 60%.

By processing the mass spectra with MALDI Biotyper software, an identification value of >2.000 was obtained for all isolates, indicating probability (2.000–2.299, 24 isolates) or high probability (2.300–3.000, nine isolates) that they belonged to the species *Lactobacillus salivarius* (Table A1). The similarity among the *L. salivarius* strains based on their MALDI-TOF mass spectra is shown in the dendrogram derived from UPGMA cluster analysis (Figure 1). At a level of similarity of ca. 60%, examined strains formed three main clusters. There was no correlation between the host and the mass profiles of the strains, although it should be noted that 5 out of 7 goose strains were grouped in cluster A. The pigeon strains were clustered in clades B and C, and the strains from chickens and turkeys were scattered across all three clades. Interestingly, the reference strains formed a separate cluster within clade A.

Analysis using the BLAST algorithm showed that the *groEL* sequences (1572 nk, GenBank Acc. No. MT862777-MT862808 and MT920907) of all strains tested were homologous to the sequences of *L. salivarius* strains deposited in the GenBank database. At least the first 30 matches (query cover > 99%) indicated *L. salivarius* species for all *groEL* sequences analysed. For 32 of the 33 tested strains, the two best matches showed an identity of ≥99.49%, and only for the Ch10a strain, a slightly lower homology, i.e., ≥98.66%, was obtained (Table A1).

### 3.2. Morphology and Growth Characteristics

**Figure 2:**
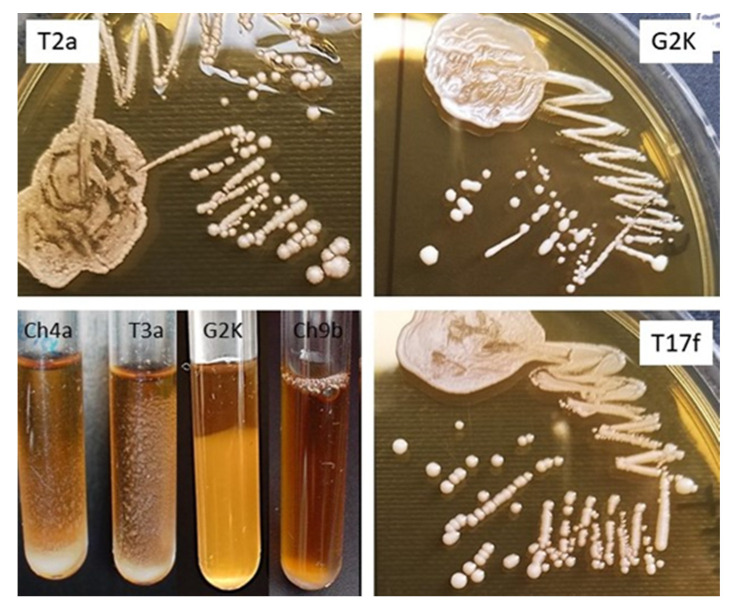
Phenotypes of selected *L. salivarius* strains on MRS agar (strains T2a, G2K, T17f) and MRS broth (strains Ch4a, T3a, G2K, Ch9b).

Most of the *L. salivarius* strains tested grew on MRS agar as large (1.2–2.2 mm), cream-colored, smooth-surfaced and brittle colonies; less frequently, the colonies were umbonate or rough with a wavy edge; several strains grew as shiny, sticky colonies (Figure 2, Table 2).

All *L. salivarius* strains, except G2K, showed the ability to grow at 45 °C, 5% CO_2_. All strains also grew under aerobic conditions at 37 °C, but the most abundant growth was recorded at 37 °C, 5% CO_2_. The growth of the strains on the broth was varied; most grew as sediment, some formed a suspension, and the cells of several strains adhered to the walls of polystyrene tubes (Figure 2). Many of the *L. salivarius* strains that grew on MRS agar as brittle colonies showed autoaggregation when grown on MRS broth (Table 2).

**Figure 3:**
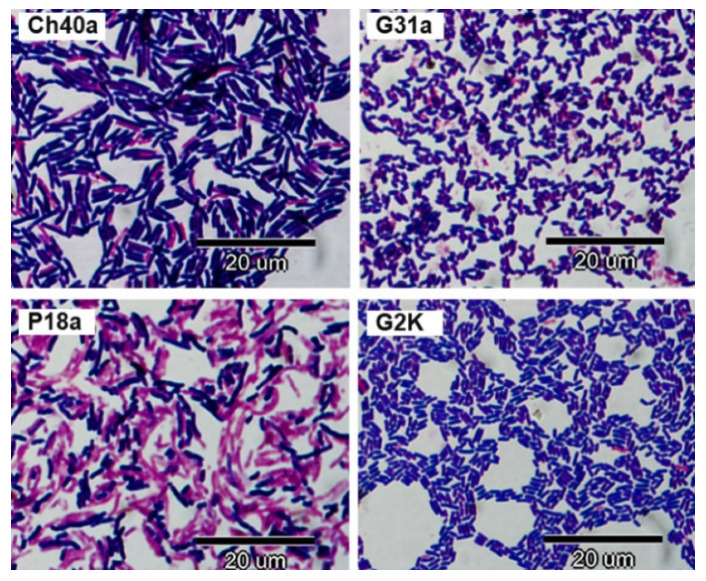
Gram staining results for selected avian *L. salivarius* strains from chicken (Ch40a), geese (G31a, G2K) and pigeon (P18a).

In the Gram method, cells of some *L. salivarius* strains stained purple, and others were Gram-labile. The cells had the form of straight or slightly bent rods varying in length from 1.8 to 7.0 µM; their thickness was 0.5 to 0.8 µM (Figure 3).

### 3.3. Fermentation Assay

**Figure 4:**
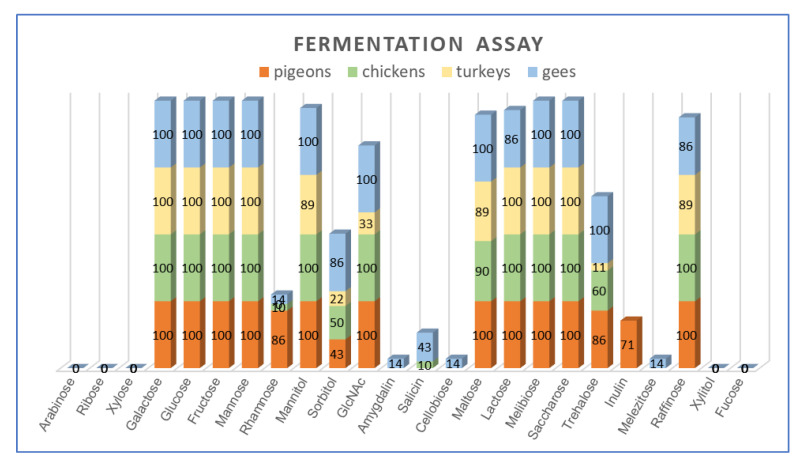
Percentage of *L. salivarius* strains capable of utilizing individual carbohydrates.

All *L. salivarius* strains were able to utilize hexoses, i.e., glucose, galactose, fructose and mannose, and disaccharides, i.e., sucrose and melibiose. As many as 97% (32/33) of the isolates grew on the medium with mannitol (except T21a) and lactose (except G2K), and 94% (31/33) of strains fermented raffinose (the exceptions were T22a and G2K). None of the strains fermented pentoses, i.e., arabinose, ribose and xylose and non-fermented xylitol or fucose. Half of the strains (16/33) showed the ability to utilize sorbitol. Substantial variation was noted among the strains for the utilization of sorbitol (48% of isolates), salicin (12%), trehalose (61%), rhamnose (24%), inulin (15%) and N-acetylglucosamine (82%) (Figure 4, Table A2).

Statistical analysis showed a significant (*p* < 0.05) relationship between the ability of *L. salivarius* strains to utilize several carbohydrates and the host. The fermentation of rhamnose and inulin was significantly more frequent in the pool of pigeon isolates compared to strains from other bird species (power of observed relations was at an average level, as indicated contingency coefficient ranged from 0.315 to 0.417). A significantly lower frequency of trehalose and N-Acetylglucosamine utilization was noted in strains from turkeys compared to isolates from pigeons (C = 0.338), chickens (C = 0.187) and geese (C = 0.420). The utilization of salicin was more frequent in geese strains compared to turkey isolates (C = 0.203).

In terms of carbohydrate utilization, the strain G2K derived from geese stood out from the rest. It was the only strain that did not ferment lactose and grew in broth supplemented with amygdalin, cellobiose and melezitose. It was also one of the two strains unable to use raffinose (Table A2). Despite this biochemical difference, the sequence analysis of 16S rDNA of the G2K strain (GenBank Acc. No. MW642195) confirmed its affiliation to the species of *L. salivarius* (identity > 99%).

### 3.4. Presence of repE, abp118β + abp118α and Genes Determining Carbohydrate Utilization

The *repE* gene, which in addition to the *repA* gene is a recognized marker of *repA*-type megaplasmids commonly found in *L. salivarius* strains [30], was detected in 31 (94%) of 33 wild-type isolates and in reference strain *L. salivarius* LMG 9477.

In the PCR with the use of primers specific for the gene encoding of the peptide alfa and beta of Abp118 bacteriocin, the PCR product was obtained for 18 (54.5%) *L. salivarius* strains, including seven isolates from turkeys, six isolates from domestic pigeons, three isolates from chicken and two isolates from geese (Table 2). The amplicon size was 410 bp for 16 strains, 390 bp for isolate T18a and 430 bp for isolate P3a (Table 3). 

Sequence analysis showed that the 410 and 390 bp amplicons corresponded to the gene encoding the Abp118 bacteriocin (identity ≥ 98%) or salivaricin P (identity ≥ 98%); however, in the case of the T18a strain (390 bp), there was a 24 consecutive nucleotide deletion in the gene encoding Abp118 alpha subunits (compared to the sequence of strain UCC118, Acc. No. AF408405.1). Within the DNA sequence of the P81, T27 and T18a amplicons, eight variable sites were detected, but they did not translate into changes in the putative amino acid sequence of bacteriocin (Table A3). The sequence (387 nk) of the 430 bp amplicon obtained for strain P3a showed 99% similarity to the gene of ABC transporter permease and bacteriocin and 98% similarity to the *blp1* and *bimlp* genes encoding putative bacteriocin subunit a and putative bacteriocin immunity protein (Table 3).

Genes associated with sugar metabolism were noted in strains containing megaplasmids as well as in one isolate (G24b) in which the *repE* gene was not detected. Despite the inability of the *L. salivarius* strains to ferment pentoses, 15% of them contained the *tktA* gene coding for transketolase, and 36% had the *mipB* gene encoding transaldolase; two strains from pigeons (P18a and P21a) had both genes which complete the pentose phosphate pathway. All rhamnose-fermenting strains contained the *rhaB* gene encoding rhamnulokinase, but it was also found in six isolates unable to grow on rhamnose-supplemented medium. Similarly, all *L. salivarius* strains utilizing sorbitol (48%), as well as three isolates (from pigeons) unable to ferment this carbohydrate, harboured the LSL_1894 gene encoding sorbitol-6-phosphate2-dehydrogenase (Table 2).

### 3.5. Phylogenetic Analysis

**Figure 5:**
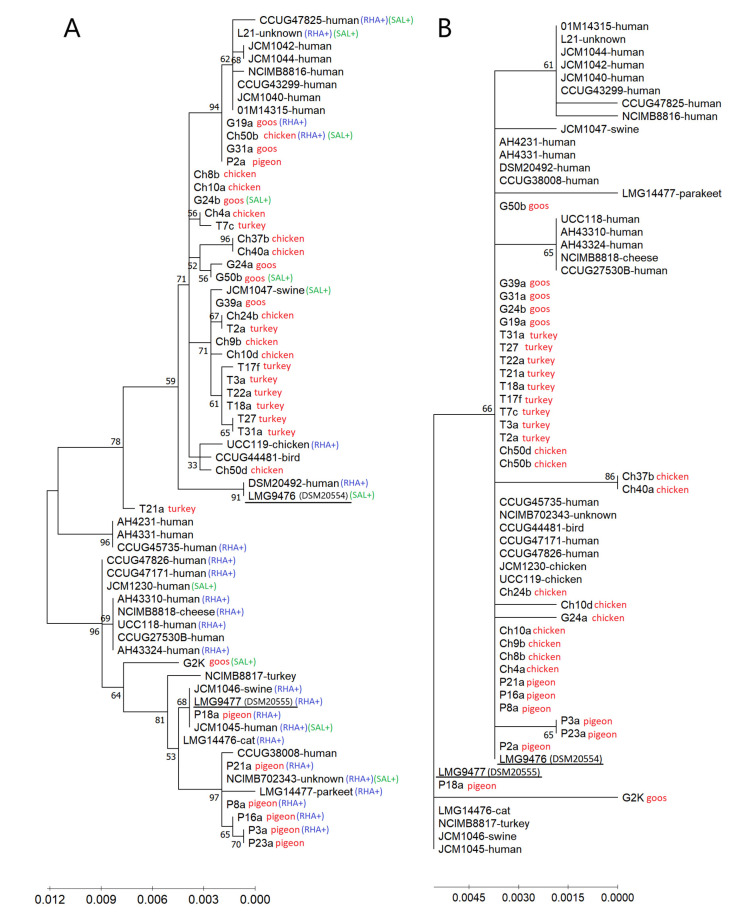
(**A**) Phylogenetic trees based on the *groEL* gene sequences of 33 wild-type avian *L. salivarius* strains tested in this study and 30 *L. salivarius* strains (mainly of human origin) previously described by Li et al. [18] built by the maximum likelihood method. The percentage of replicate trees in which the associated taxa were clustered together in the bootstrap test (500 replicates) is shown next to the branches. Scale bars show genetic distance; RHA+ and SAL+ indicate the ability of strains to utilize rhamnose and salicin, respectively. (**B**) Dendrogram showing the similarity among the predicted amino acid sequences of the GroEL chaperonin built by the maximum likelihood method.

In the dendrogram resulting from the comparative analysis of the *groEL* sequence of wild-type isolates tested in this study and 30 additional *L. salivarius* strains (21 strains from humans and nine strains from animals or other sources), previously characterized by Li et al. [17], two reference strains, LMG 9476 (formerly subspecies *salicinus*) and LMG 9477 (formerly subspecies *salivarius*), were separated into distinct branches, each of which comprised a mixture of strains (Figure 5A).

The LMG 9477 strain formed a common clade together with the pigeon-derived *L. salivarius* strains (except for the P2a strain) and with the goose-derived G2K strain. Most pigeon strains and human strains of this clade showed the ability to ferment rhamnose. However, several other strains (including wild-type strains Ch50b and G19a) with the ability to utilize this carbon source were clustered in a second major clade containing the LMG 9476 strain. Strains capable of utilizing salicin were scattered in both clades. The clade containing *L. salivarius* LMG 9476 was further split into several clusters, one of which grouped the majority (6 of 9) of turkey isolates (T3a, T17f, T18a, T22a, T27 and T31a). Human strains were largely in separate clusters, within both the LMG 9476 clade and the LMG 9477 clade, while single reference animal strains (from birds and swine) were generally grouped with the wild-type isolates tested in this study (Figure 5A). This indicates a phylogenetic distance between *L. salivarius* strains from humans and birds.

The significant differences within the *groEL* gene sequence did not translate into differences in the amino acid sequence. The predicted amino acid sequences of GroEL chaperonin were identical for 25 out of 33 isolates tested in this study and 11 other strains (mainly of human origin); they clustered together in the dendrogram with the *L. salivarius* LMG 9476 strain (formerly subsp. *salicinus*) (Figure 5B). Interestingly, in the *groEL* gene of the Ch10d strain (Acc. No. MT862788), a deletion of as many as 18 consecutive nucleotides was noted, corresponding to eight amino acids.

The G + C content in the *groEL* gene of avian strains grouped with the LMG 9477 strain ranged from 36.22 (P3a and P23a) to 36.64 mol% (G2K), and the average content of G + C in this clade was 36.36 mol%. In the avian strains forming the second main clade (containing the LMG 9476 strain), the mean G + C content was slightly lower, i.e., 36.21 mol%, and ranged from 35.96 (strain Ch24b) to 36.47 mol% (T21a).

### 3.6. Tolerance to Bile

All isolates tested were able to survive for 24 h in the presence of 2% bile, and 73% of them even grew in the MRS broth supplemented with 1% or 2% ox gall. However, the growth of the vast majority of isolates was considered poor (3.5%–17% compared to the positive control), and only two strains, G2K (100% growth) and Ch40a (24%–38%), showed intensive growth on bile medium (Table 2). There were no significant differences in growth intensity in broth supplemented with 1% and 2% bile (Table A4), but most strains showed more intense growth on bile medium after 48 h compared to the 24-h culture (Table 2). A significantly higher frequency of strains capable of growing in the presence of 2% bile was recorded in the pool of pigeon and goose isolates compared to strains from chickens and turkeys (C 0.205–2.39) after 48 h of incubation. After 24 h of incubation, a significantly higher frequency of growth was recorded only in the pool of pigeon isolates compared to turkey strains (C = 0.264).

### 3.7. Hydrophobicity

The vast majority (82%) of the *L. salivarius* strains tested were hydrophobic, and for as many as 68% of the strains, including all turkey isolates, the %H value was >95%. Six isolates, including two (28%) strains from pigeons, one (10%) strain from chickens and three (43%) strains from geese, were classified as hydrophilic; the extremely low value of %H = 0 was recorded for two strains, i.e., Ch50b and G2K. It is worth noting that all strains showing low or moderate hydrophobicity, i.e., 0%–55%, were able to grow in broth containing 1% or 2% bile (Table 2).

### 3.8. Phenotypic and Genotypic Profiles of Antimicrobial Resistance

The phenotypic and genotypic drug resistance profiles of some *L. salivarius* strains tested in this work, i.e., those from pigeons and turkeys, were taken from our previous papers [5,6] (Table 4).

In the pool of studied isolates, antibiotic-resistant strains were found with high frequency, and as many as 51% of them were multiresistant (resistance to at least three groups of antimicrobial agents). Resistance to streptomycin, kanamycin, enrofloxacin and tetracycline was reported most frequently (>40% of strains), and high MIC values for ampicillin were confirmed in several isolates from turkeys. Strains from pigeons, chickens and turkeys showed the highest frequency of resistance, while goose isolates were susceptible to all antibiotics, except for aminoglycosides. Only one strain, i.e., G24a, was susceptible to all antimicrobial agents (Table 4).

Phenotypic antibiotic resistance was correlated with the presence of resistance genes. *TetL* and *tetM*, *ermB* and *lnuA* genes were widespread in pigeon, chicken and turkey strains, and other resistance genes were less frequently detected. None of the considered resistance genes were detected in 12 *L. salivarius* strains, including all strains from geese (Table 4).

## 4. Discussion

Among the main goals of this research was to evaluate the fermentation profiles of avian *L. salivarius* strains. Historically, the ability to ferment rhamnose or salicin was considered to be a criterion distinguishing *L. salivarius* subsp. *salivarius* from *L. salivarius* subsp. *salicinius*. However, subsequent polyphasic analyses by Li et al. [18] showed that the initially proposed taxonomic criteria were insufficient, and ultimately, these two subspecies were unified into a single species. The validity of this unification was also confirmed by the results of this study, as most of the avian *L. salivarius* strains were unable to utilize rhamnose (utilized by 24% strains) and salicin (utilized by 12% strains). Moreover, one isolate (Ch50b) showed the ability to utilize both of these carbohydrates. Our results differ significantly from those of Li et al. [18], who showed the fermentation of rhamnose and salicin by 57% and 27% of *L. salivarius* strains (mainly of human origin), respectively. The ability of all *L. salivarius* isolates in the present study to ferment glucose, galactose, fructose, mannose and sucrose, as well as their inability to utilize arabinose and xylose, is fully consistent with the previous study by Li et al. [18]. However, contrary to our results, these authors reported the utilization of D-ribose by several (8/30) *L. salivarius* strains. Although sorbitol-fermenting ability was originally considered a characteristic feature of the species *L. salivarius* [1], more than half of the avian strains were unable to utilize this carbohydrate. Our results differ significantly from the reports of Li et al. [18], who noted sorbitol fermentation in 88% of strains in a pool of human isolates. Even higher values are indicated by the API 50CHL test guidelines [34], according to which as many as 98% of *L. salivarius* strains are able to utilize sorbitol.

The correlation recorded in the present study among the utilization of certain carbohydrates (rhamnose, inulin, NAG, trehalose and salicin) by *L. salivarius* strains and the host indicates the evolutionary variability of *L. salivarius* bacteria. The phenomenon of the evolution of commensal bacteria in accordance with the nutritional conditions of the host environment has long been widely recognized [35,36], and Lee et al. [8] demonstrated that among the host-specific genes detected in *L. salivarius* strains, several are associated with nutrient utilization. The diversity in the biochemical profiles across tested *L. salivarius* strains may be the result of the variability of the genome within the *repA*-type megaplasmids [20], where several genes related to sugar metabolism (*mipB*, *tktA*, *rhaB* and LSL_1894) are located [30]. The presence of the rhamnulokinase gene was detected not only in *L. salivarius* strains fermenting rhamnose but also in strains without this ability, which has previously been demonstrated by Li et al. [30] and points to gene silencing or a lack of other genes that are essential for completing the pathway. The same authors also reported the prevalence of genes encoding transaldolase (*mipB*) and transketolase (*tktA*) in human *L. salivarius* strains lacking ribose utilization. This study is the first report indicating the possibility of the utilization of cellobiose and melezitose as carbon sources by *L. salivarius* strains [1,18]. It is also worth emphasizing that inulin, which is utilized by *L. salivarius* strains from pigeons, is a long-chain polysaccharide that acts as a prebiotic by selectively stimulating the growth and activity of beneficial intestinal microbiota [37]. The utilization of inulin-type fructans is common in bifidobacteria, while in lactobacilli, the degradation of this polysaccharide is rare [38].

We showed that *groEL* sequences can be used not only for phylogenetic analyses but also for the identification of *L. salivarius* strains. Several authors have demonstrated that the *groEL* gene can be successfully used to identify a variety of Gram-positive bacteria, including lactobacilli [39], enterococci [40] and bifidobacteria [41]. Contrary to earlier reports [18], we showed a certain correlation between *groEL*-based phylogenesis and the host and biochemical profiles of the isolates tested. The slightly higher mean G + C content in the *groEL* sequences of *L. salivarius* strains grouped with the reference strain LMG 9477 (DSM 20555T subs. *salivarius*) than the G + C content in the LMG9477 clade is consistent with the earlier results of Li et al. [18].

In this study, we demonstrated large diversity among avian strains of *L. salivarius* in terms of colony morphology, growth characteristics on broth (sediment, suspension formation and autoaggregation ability) and biofilm formation, as well as hydrophobicity and bile resistance. These features may be dependent on the production of extracellular polysaccharides (EPS) by bacteria [42,43,44,45]. EPS are thought to protect bacterial cells against extreme conditions (temperature, light intensity, pH or osmotic stress) or biotic stress [43], and they can be either weakly or strongly bound to the bacterial cell surface [46]. EPS of lactobacilli are involved in cell adhesion/recognition mechanisms and could confer a range of local and systemic health benefits to the host, including immune modulation, lowering blood cholesterol or stimulation of the growth of beneficial gut microbiota [47,48].

Several authors have reported that EPS-rich *Lactobacillus* strains grow as slimy colonies on agar and form a suspension on broth [43,44]. EPS-poor strains form sediment at the bottom of the tube as they grow on the broth. It, therefore, appears that the growth characteristics of *L. salivarius* bacteria on both agar and broth allow for the initial differentiation of EPS-rich and EPS-poor strains.

Biofilm formation by probiotic bacteria is considered a beneficial property, as it may allow them to colonize the intestinal epithelium while displacing unfavourable microflora. The positive correlation recorded in this study between the ability to self-aggregate and biofilm formation has previously been noted for other *Lactobacillus* species [49]. Autoaggregation is mediated by surface autoagglutinins, mainly proteins, and is often among the first steps in forming biofilms [50]. Strong biofilm formation by strains with brittle colonies, which are likely to have poor EPS production, is consistent with the results of Tsuneda et al. [45], who showed that EPS-poor strains adhere more strongly to a glass surface than EPS-rich strains. Moreover, they showed that the EPS of EPS-poor strains contain twice as much protein as the EPS of EPS-rich strains.

Several researchers have reported a positive correlation between the hydrophobicity of *Lactobacillus* strains and their adhesion to epithelial cells [7,51]. In our study, we did not find a clear relationship between the hydrophobicity of *L. salivarius* strains and their biofilm formation capacity or colony morphology. This is in line with reports by Tsuneda et al. [45], who found no correlation between hydrophobicity and cell adhesiveness for either EPS-rich or EPS-poor strains.

Bile tolerance is among the most crucial properties for probiotic bacteria, as it determines their ability to survive transit through the duodenum. Bacterial resistance to bile is mediated by bile salt hydrolysing enzymes (BSH) and by EPS, which acts as a protective coating against unfavourable environmental factors [42,52]. A positive correlation between EPS production and bile tolerance was recorded in bifidobacteria [42], but in the case of lactobacilli, the relationship between EPS and bile resistance remains unclear [53]. We found no relationship between colony structure or broth growth characteristics (which are dependent on EPS production) and bile tolerance, and, therefore, it seems that EPS do not play a leading role in determining bile resistance in *L. salivarius* strains. The tendency observed in this study of increased bile tolerance of *L. salivarius* strains over time is consistent with several previous reports [53,54].

Bacteriocinogenic non-pathogenic strains are desirable probiotics. The production of the bacteriocin Abp118, the gene of which we detected in most strains tested in this study, has previously been confirmed in many human *L. salivarius* strains [30]. It is a class IIb heat-stable dipeptide bacteriocin that shows a high homology to salivaricin P (they differ only in two amino acids) [55]. Both bacteriocins inhibit the growth of sensitive strains of *Listeria monocytogenes*, *Enterococcus* sp., *Lactobacillus* sp., *Pediococcus* sp., *Lecuconostoc* sp. and *Streptococcus* sp. [55]. Bases on these results, it can be assumed that abp118-positive *L. salivarius* strains inhabiting the gastrointestinal tract of birds contribute to competitive exclusion and modulate the intestinal microbiota. It should be noted, however, that not all *L. salivarius* strains containing genes encoding the Abp118 bacteriocin show antibacterial activity in vitro [30]. Therefore, further studies are needed to determine the expression of the *abp118alpha* and *abp118beta* genes in avian *L. salivarius* strains and the related antimicrobial activity.

The issue of antibiotic resistance of *Lactobacillus* bacteria from poultry and domestic pigeons, including *L. salivarius* strains, was widely discussed in our previous papers [5,6,56,57]. Due to drug resistance, most *L. salivarius* strains from poultry and domestic pigeons are disqualified as potential probiotics. Only strains showing drug sensitivity and no resistance genes can be used as microbial feed additives [33]. Despite the beneficial effects of *L. salivarius* in birds [25], consideration should also be given to the risk of possible transmission of resistance genes between them and other members of the intestinal microbiota, including potentially pathogenic bacteria. Many resistance genes detected in this study have previously been found in lactobacilli and other lactic acid bacteria on mobile elements [5,6], and the conjugative Tn916/Tn1545-like transposon has been identified in one *L. salivarius* strain (T22a).

## 5. Conclusions

The results of the phenotypic and genotypic analyses carried out in this study broaden the knowledge on the species *L. salivarius* and may be useful both in determining taxonomic affiliation and in the selection of probiotic strains. Due to the phenotypic diversity of avian *L. salivarius*, including the presumed production of Abp118 bacteriocin and differentiation in EPS production, individual strains can be assumed to have varying abilities to colonize distinct niches and different effects on the host. The host-specific traits of the *L. salivarius* strains indicate their evolutionary adaptation and confirm the principle promoted by scientists that, due to biological compatibility, probiotics intended for a given host species should be derived from its natural microflora.

## Figures and Tables

**Table 1:** Primers used in this study.

Gene	Encoded Protein	Primers	Annealing Temp. [°C]	PCR Product [bp]
*repE*	Hypothetical replication protein	F: ATGAAAAGTCTTACATCTCGTG R: TTAGAAACTCAATAATACGTTTAATTC	54	993
*abp 118β + abp 118α*	Abp118*β* + *α* bacteriocin peptide	F: AAGGAATTTACAGTATTGACAG R: ACGGCAACTTGTAAAACCA	53	390–410
*rhaB*	Rhamnulokinase	F: TTAGGAATTGATACTTGGGC R: ATCCGCCACCAACTATATTC	54	990
LSL-1894	Sorbitol-6-phosphate 2-dehydrogenase	F: ATGAGTGAGAACTGGCTG R: TCCGCGAGATTTTCCTCC	54	801
*mipB*	Transaldolase	F: ATGGAATTTTTATTAGATACAGTTG R: CTATAAGTTATTTATATTTTTGTCAC	52	681
*tktA*	Transketolase	F: ATGTATGATCAAGTAGACC R: TTATTTTTCCAAATATTTATCAACG	52	1992

**Table 2:** *L. salivarius* strains used in this study and their colony morphology, broth growth characteristics, biofilm formation capacity, hydrophobicity and tolerance to 2% bile; capability of the strains to utilize arabinose (ARA), ribose (RIB) and xylose (XYL), rhamnose (RHA) and sorbitol (SOR). Distribution of the *repE*, bacteriocin Abp118 genes and genes related to sugar metabolism (*mipB*, *tktA*, *rhaB* and LSL_1894). ND*—the structure of the colonies was not determined as they were neither clearly brittle nor clearly sticky; the meaning of the signs “−”, “+”, “++” and “+++” are explained in the Section 2.

Isolate or Reference Strain	Isolation Host	Colony Morphology	Colony Structure	Growth on MRS Broth		Biofilm	Hydrophobicity	Growth on MRS + 2% Bile				Pentose Utilization			Rhamnose		Sorbitol	
				Suspension	Autoaggregation			24 h	48 h	*abp 118α + β*	*repE*	ARA, RIB, XYL	*mipB*	*tktA*	RHA	*rhaB*	SOR	LSL_1894
LMG 9476	Human	convex, smooth	ND*	−	−	+	100%	<3%	6%	−	−	−	+	−	−	−	+	+
LMG 9477	Human	convex, smooth	brittle	+	−	+	100%	<3%	4%	−	+	−	+	−	+	+	+	+
P2a	Pigeon	convex, smooth	ND	−	−	++	10%	9%	11%	−	+	−	−	−	+	+	−	−
P3a	Pigeon	umbonate, smooth	sticky	++	−	+++	100%	8%	9%	−	+	−	−	−	+	+	−	+
P8a	Pigeon	convex, smooth	brittle	−	−	++	100%	<3%	7%	+	+	−	+	−	+	+	−	+
P16a	Pigeon	convex, smooth	ND	−	−	+	74%	4%	12%	+	+	−	−	−	+	+	+	+
P18a	Pigeon	convex, smooth	sticky	+	−	++	10%	8%	8%	+	+	−	+	+	+	+	+	+
P21a	Pigeon	umbonate, smooth	brittle	−	−	+++	100%	<3%	6%	+	+	−	+	+	+	+	−	+
P23a	Pigeon	convex, smooth	ND	−	−	+++	100%	6%	6%	+	+	−	−	−	−	−	+	+
Ch4a	Chicken	convex, smooth	brittle	−	++	+++	100%	<3%	5%	+	+	−	−	−	−	−	−	−
Ch8b	Chicken	convex, smooth	brittle	+	−	+	67%	<3%	<3%	−	+	−	−	−	−	−	−	−
Ch9b	Chicken	convex, smooth	ND	−	−	+	60%	<3%	<3%	−	+	−	−	−	−	+	−	−
Ch10a	Chicken	convex, rough, undulate	brittle	−	++	+++	100%	<3%	<3%	+	+	−	+	−	−	−	+	+
Ch10d	Chicken	umbonate, smooth	brittle	−	++	+++	100%	<3%	<3%	−	+	−	−	−	−	−	+	+
Ch24b	Chicken	umbonate, rough, undulate	brittle	−	++	+++	100%	<3%	<3%	+	+	−	−	−	−	−	−	−
Ch37b	Chicken	umbonate, smooth	ND	++	−	++	100%	3.5%	12%	−	+	−	−	−	−	−	+	+
Ch40a	Chicken	umbonate, smooth	ND	+	−	+	55%	24%	37%	−	+	−	+	−	−	−	+	+
Ch50b	Chicken	convex, smooth	sticky	−	−	+	0%	5%	10%	−	+	−	+	−	+	+	+	+
Ch50d	Chicken	convex, smooth	brittle	−	++	+++	100%	5%	4.5%	−	+	−	−	+	−	−	−	−
T2a	Turkey	convex, rough, undulate	brittle	−	++	++	100%	4%	4%	−	+	−	−	−	−	+	−	−
T3a	Turkey	convex, rough, undulate	brittle	−	++	+++	100%	8%	6%	+	+	−	−	+	−	+	−	−
T7c	Turkey	convex, smooth	sticky	+	−	++	100%	<3%	<3%	+	+	−	−	+	−	−	−	−
T17f	Turkey	convex, smooth	brittle	−	+	+	100%	<3%	<3%	−	−	−	−	−	−	−	−	−
T18a	Turkey	convex, rough	brittle	−	−	+	95%	<3%	<3%	+	+	−	+	−	−	−	+	+
T21a	Turkey	convex, smooth	brittle	−	++	+++	100%	<3%	<3%	+	+	−	−	−	−	+	−	−
T22a	Turkey	convex, smooth	ND	+	−	++	100	<3%	<3%	+	+	−	−	−	−	−	−	−
T27	Turkey	convex, smooth	brittle	+	−	++	100	<3%	3.5%	+	+	−	−	−	−	−	−	−
T31a	Turkey	convex, smooth	ND	++	−	++	100	<3%	6%	+	+	−	+	−	−	−	+	+
G2K	Goose	convex, smooth	sticky	++	−	+	0	100%	100%	−	+	−	+	−	−	−	+	+
G19a	Goose	convex, smooth	brittle	+	−	+	100	13%	17%	+	+	−	−	−	+	+	−	−
G24a	Goose	convex, smooth	brittle	+	−	+	13	13%	16%	−	+	−	+	−	−	+	+	+
G24b	Goose	convex, rough, undulate	brittle	−	++	+++	100	9%	10%	−	−	−	+	−	−	−	+	+
G31a	Goose	convex, smooth	sticky	++	−	+	100	13%	17%	+	+	−	−	−	−	+	+	+
G39a	Goose	convex, rough	brittle	−	++	-	100	<3%	7%	−	+	−	+	−	−	+	+	+
G50b	Goose	convex, smooth	sticky	−	+	+++	42	7%	13%	−	+	−	−	−	−	−	+	+
Total: 33 [%]										17 [51%]	31 [94%]	0	12 [36%]	5 [15%]	8 [24%]	15 [45%]	16 [48%]	19 [57%]

**Table 3:** BLAST results of sequence analysis of *abp118 a* + *abp118 β* bacteriocin amplicons.

Strain	PCR Product [bp]	Length of Sequence Deposited in GenBank; Acc. No.	% of Similarity; Sequence ID (GenBank)	
P3a	430	387 nk MW478293	99%—*L. salivarius* strain ZLS006 plasmid unnamed1; CP020859.1	<1…174 nk—ABC transporter permease 191…>385 nk—bacteriocin
			98%—*L. salivarius* strain BGHO1 plasmid pMPHO1; JQ322756.1; q.c. 97%	<1…195 nk—gene *blp1a* -putative bacteriocin subunit a 212…>387nk—gene *bimlp*—putative bacteriocin immunity protein
P8a & T27	410	368 nk MW478294 MW478296	98%—*L. salivarius* salivaricin CRL1328 gene cluster; EF592482.1	<1…150 nk—salivaricin CRL1328 alpha peptide 168…>368 nk—salivaricin CRL1328 beta peptide
			98%—*L. salivarius* UCC118 plasmid pMP118; CP000234.1	<1…202 nk—*abp118β*—Abp118 bacteriocin *β* peptide 219..>368 nk—*abp118a—*Abp118 bacteriocin *a* peptide
			98%—*L. salivarius* subsp. *salivarius* bacteriocin-like prepeptides, strain UCC118; AF408405.1	<1…150 nk—*abp118alpha* gene—Abp118 alpha 168…>368 nk *abp118beta* gene—Abp118 beta
T18a	390	344 nk (deletion of 24 nk) MW478297	93%—*L. salivarius* salivaricin CRL1328 gene cluster; EF592482.1	<1…150 nk—salivaricin CRL1328 alpha peptide 168…>368 nk- salivaricin CRL1328 beta peptide
			93%—*L. salivarius* UCC118 plasmid pMP118; CP000234.1	<1…201 nk—*abp118b*—Abp118 *β* peptide 219...>368 nk- *abp118a—*Abp118 *a* peptide
			93%—*L. salivarius* subsp. *salivarius* bacteriocin-like prepeptides, strain UCC118; AF408405.1	<1…150 nk—*abp118alpha* -Abp118 alpha 168…>368 nk—*abp118beta -*Abp118 beta

**Table 4:** Phenotypic and genotypic antibiotic resistance profiles of *Ligilactobacillus salivarius* strains.

	Isolate	Phenotypic Antibiotic Resistance									Resistance Genes and Integrase Gene									
pigeons	P2a		TET	KAN							*tetL*									
	P3a		TET	KAN	STR			ENR			*tetL*	*tetM*								
	P8a			KAN	STR						*tetL*									
	P16a		TET	KAN	STR			ENR			*tetL*	*tetM*								
	P18a			KAN	STR								*lnuA*							
	P21a			KAN	STR															
	P23a		TET	KAN			CHL	ENR	LIN	ERY	*tetL*	*tetM*	*lnuA*	*cat*	*ermB*					
chickens	Ch4a			ND	STR				LIN							*ermC*		*ant(6)-Ia*	*lsaE*	
	Ch8b			KAN			CHL	ENR				*tetM*								
	Ch9b			KAN																
	Ch10a		TET	ND	STR				LIN	ERY	*tetL*	*tetM*			*ermB*					
	Ch10d		TET	ND			CHL	ENR	LIN	ERY	*tetL*	*tetM*			*ermB*					
	Ch24b		TET	ND	STR	GEN	CHL	ENR	LIN	ERY	*tetL*	*tetM*	*lnuA*		*ermB*		*bif*			
	Ch37b		TET	ND									*lnuA*			*ermC*				
	Ch40a			ND				ENR	LIN	ERY			*lnuA*	*cat*	*ermB*					
	Ch50b			KAN	STR		CHL													
	Ch50d			KAN	STR									*cat*						
turkeys	T 2a			ND				ENR												
	T 3a		TET	ND				ENR	LIN	ERY	*tetL*	*tetM*			*ermB*	*ermC*				
	T 7c			ND	STR			ENR	LIN				*lnuA*					*ant(6)-Ia*	*lsaE*	
	T 17f	AMP	TET	ND				ENR	LIN	ERY	*tetL*	*tetM*			*ermB*					
	T 18a	AMP	TET	ND			CHL	ENR	LIN	ERY	*tetL*	*tetM*	*lnuA*		*ermB*					
	T 21a	AMP	TET	ND			CHL	ENR	LIN	ERY	*tetL*	*tetM*								
	T 22a		TET	ND	STR	GEN	CHL	ENR	LIN	ERY	*tetL*	*tetM*			*ermB*					*int-Tn*
	T 27			ND	STR			ENR												
	T 31a	AMP	TET	ND				ENR			*tetL*	*tetM*								
gees	G 2K			KAN	STR															
	G 19a			KAN																
	G 24a																			
	G 24b			ND	ND	ND	ND													
	G 31a			ND	ND	ND	ND													
	G 39a			KAN	STR															
	G 50b			KAN	STR															
		12%	42%	ND	48%	6%	24%	48%	36%	30%	42%	39%	21%	9%	27%	12%	3%	6%	3%	6%

AMP, ampicillin; CHL, chloramphenicol; CLIN, clindamycin; ENR, enrofloxacin; ERY, erythromycin; GEN, gentamycin; KAN, kanamycin; LIN, lincomycin; STR, streptomycin; TET, tetracycline; bif, bifunctional *aac(6′)Ie-aph(2″)Ia* gene.

## Data Availability

Nucleotide sequences reported in this paper are available in the NCBI GenBank database under the following accession numbers: MT862775-MT862808 and MT920907 (*groEL* gene), MW478293-MW478294 and MW478296-MW478297 (genes coding for bacteriocin), MW642195 (16S rDNA of the G2K strain).

## Appendix A

**Table A1:** Identification of *L. salivarius* strains by MALDI-TOF mass spectrometry, *groEL* sequence analysis and analysis of 16S rDNA or 16S-23S rDNA regions.

Strain	Identification by MALDI-TOF MS		Identification Based on Sequence Analysis of the *groEL* Gene			Analysis of 16S rDNA or 16S-23S rDNA
		*groEL* Acc. No.	Two Best Matches; GenBank Accesion Number	Query Cover [%]	Identity [%]	
P2a	*L. salivarius* 2.393	MT862777	Ligilactobacillus salivarius strain 2D; CP047412.1	100	99.94	ND
			Lactobacillus salivarius str. Ren Select; CP011403.1	100	99.87	
P3a	*L. salivarius* 2.362	MT862778	Lactobacillus salivarius CECT 5713; CP002034.1	100	99.81	ND
			Lactobacillus salivarius strain NCIMB702343 groEL; DQ444339.1	99	99.81	
P8a	*L. salivarius* 2.050	MT862779	Lactobacillus salivarius CECT 5713; CP002034.1	100	99.94	ND
			Lactobacillus salivarius strain NCIMB702343 groEL; DQ444339.1	99	99.94	
P16a	*L. salivarius* 2.367	MT862780	Lactobacillus salivarius CECT 5713; CP002034.1	100	99.94	ND
			Lactobacillus salivarius strain NCIMB702343 groEL; DQ444339.1	100	99.94	
P18a	*L. salivarius* 2.228	MT862781	Ligilactobacillus salivarius strain BCRC 14759; CP024067.1	100	99.94	ND
			Ligilactobacillus salivarius strain ZLS006; CP020858.1	100	99.94	
P21a	*L. salivarius* 2.023	MT862782	Lactobacillus salivarius CECT 5713; CP002034.1	100	99.94	ND
			Lactobacillus salivarius strain NCIMB702343 groEL; DQ444339.1	99	99.94	
P23a	*L. salivarius* 2.322	MT862783	Lactobacillus salivarius CECT 5713; CP002034.1	100	99.81	ND
			Lactobacillus salivarius strain NCIMB702343 groEL; DQ444339.1	99	99.81	
Ch4a	*L. salivarius* 2.118	MT862784	Lactobacillus salivarius strain CCUG44481 groEL; DQ444352.1	100	99.75	ND
			Ligilactobacillus salivarius strain CICC 23174; CP017107.1	100	99.68	
Ch8b	*L. salivarius* 2.168	MT862785	Lactobacillus salivarius strain CCUG44481 groEL; DQ444352.1	100	99.81	*L. salivarius* [3,28]
			Ligilactobacillus salivarius strain CICC 23174; CP017107.1	100	99.75	
Ch9b	*L. salivarius* 2.264	MT862786	Lactobacillus salivarius strain CCUG44481 groEL; DQ444352.1	100	99.87	ND
			Ligilactobacillus salivarius strain CICC 23174; CP017107.1	100	99.81	
Ch10a	*L. salivarius* 2.006	MT862787	Lactobacillus salivarius strain CCUG44481 groEL; DQ444352.1	100	99.87	ND
			Ligilactobacillus salivarius strain CICC 23174; CP017107.1	100	99.81	
Ch10d	*L. salivarius* 2.249	MT862788	Lactobacillus salivarius strain CCUG44481 groEL; DQ444352.1	100	98.66	*L. salivarius* [3,28]
			Ligilactobacillus salivarius strain CICC 23174; CP017107.1	100	98.60	
Ch24b	*L. salivarius* 2.080	MT862789	Lactobacillus salivarius strain CCUG44481 groEL; DQ444352.1	100	99.68	*L. salivarius* [3,28]
			Ligilactobacillus salivarius strain CICC 23174; CP017107.1	100	99.62	
Ch37b	*L. salivarius* 2.324	MT862790	Lactobacillus salivarius strain CCUG44481 groEL; DQ444352.1	100	99.43	ND
			Ligilactobacillus salivarius strain CICC 23174; CP017107.1	100	99.43	
Ch40a	*L. salivarius* 2.001	MT920907	Lactobacillus salivarius strain CCUG44481 groEL; DQ444352.1	100	99.62	ND
			Ligilactobacillus salivarius strain CICC 23174; CP017107.1	100	99.55	
Ch50b	*L. salivarius* 2.241	MT862791	Ligilactobacillus salivarius strain 2D; CP047412.1	100	100	*L. salivarius* [3,28]
			Lactobacillus salivarius str. Ren Select; CP011403.1	100	99.94	
Ch50d	*L. salivarius* 2.124	MT862792	Lactobacillus salivarius strain CCUG44481 groEL; DQ444352.1	100	99.75	ND
			Ligilactobacillus salivarius strain CICC 23174; CP017107.1	100	99.55	
T2a	*L. salivarius* 2.010	MT862793	Lactobacillus salivarius strain CCUG44481 groEL; DQ444352.1	100	99.81	ND
			Ligilactobacillus salivarius strain CICC 23174; CP017107.1	100	99.75	
T3a	*L. salivarius* 2.164	MT862794	Lactobacillus salivarius strain CCUG44481 groEL; DQ444352.1	100	99.81	ND
			Ligilactobacillus salivarius strain CICC 23174; CP017107.1	100	99.75	
T7c	*L. salivarius* 2.002	MT862795	Lactobacillus salivarius strain CCUG44481 groEL; DQ444352.1	100	99.75	ND
			Ligilactobacillus salivarius strain CICC 23174; CP017107.1	100	99.68	
T17f	*L. salivarius* 2.311	MT862796	Ligilactobacillus salivarius strain CICC 23174; CP017107.1	100	99.81	ND
			Lactobacillus salivarius strain CCUG44481 groEL; DQ444352.1	100	99.75	
T18a	*L. salivarius* 2.390	MT862797	Lactobacillus salivarius strain CCUG44481 groEL; DQ444352.1	100	99.81	ND
			Ligilactobacillus salivarius strain CICC 23174; CP017107.1	100	99.75	
T21a	*L. salivarius* 2.003	MT862798	Lactobacillus salivarius strain CCUG44481 groEL; DQ444352.1	100	99.49	ND
			Ligilactobacillus salivarius strain CICC 23174; CP017107.1	100	99.43	
T22a	*L. salivarius* 2.136	MT862799	Lactobacillus salivarius strain CCUG44481 groEL; DQ444352.1	100	99.81	ND
			Ligilactobacillus salivarius strain CICC 23174; CP017107.1	100	99.75	
T27	*L. salivarius* 2.400	MT862800	Lactobacillus salivarius strain CCUG44481 groEL; DQ444352.1	100	99.75	ND
			Ligilactobacillus salivarius strain CICC 23174; CP017107.1	100	99.68	
T31a	*L. salivarius* 2.241	MT862801	Lactobacillus salivarius strain CCUG44481 groEL; DQ444352.1	100	99.68	ND
			Ligilactobacillus salivarius strain CICC 23174; CP017107.1	100	99.62	
G2K	*L. salivarius* 2.106	MT862802	Lactobacillus salivarius strain JCM1230 groEL; DQ444335.1	100	99.55	*L. salivarius* [3,28]
			Lactobacillus salivarius isolate LPM01 genome; LT604074.1	100	99.49	
G19a	*L. salivarius* 2.139	MT862803	Ligilactobacillus salivarius strain 2D; CP047412.1	100	100	*L. salivarius* [3,28]
			Lactobacillus salivarius str. Ren Select; CP011403.1	100	99.94	
G24a	*L. salivarius* 2.273	MT862804	Lactobacillus salivarius strain CCUG44481 groEL; DQ444352.1	100	99.68	*L. salivarius* [3]
			Ligilactobacillus salivarius strain CICC 23174; CP017107.1	100	99.62	
G24b	*L. salivarius* 2.305	MT862805	Lactobacillus salivarius strain CCUG44481 groEL; DQ444352.1	100	99.87	*L. salivarius* [3]
			Ligilactobacillus salivarius strain CICC 23174; CP017107.1	100	99.81	
G31a	*L. salivarius* 2.211	MT862806	Ligilactobacillus salivarius strain 2D; CP047412.1	100	100	*L. salivarius* [3]
			Lactobacillus salivarius str. Ren Select; CP011403.1	100	99.94	
G39a	*L. salivarius* 2.029	MT862807	Lactobacillus salivarius strain CCUG44481 groEL; DQ444352.1	100	99.81	*L. salivarius* [3]
			Ligilactobacillus salivarius strain CICC 23174; CP017107.1	100	99.75	
G50b	*L. salivarius* 2.135	MT862808	Lactobacillus salivarius strain CCUG44481 groEL; DQ444352.1	100	99.75	*L. salivarius* [3]
			Ligilactobacillus salivarius strain CICC 23174; CP017107.1	100	99.68	

**Table A2:** Carbohydrate fermentation profiles of *L. salivarius* strains: ARA—arabinosel; RIB—ribose; XYL—xylose; GAL—galactose; GLU—glucose; FRU—fructose; MNE—mannose; RHA—rhamnose; MAN—mannitol; SOR—sorbitol; NAG-N—acethylglucosamine; AMY—amygdalin; SAL—salicin; CEL—cellobiose; MAL—maltose; LAC—lactose; MEL—melibiose; SAC—sacharose; TRE—trehalose; INU—inulin; MLZ—Melezitose; RAF—raffinose; XLT—xylitol; FUC—fucose. Symbols indicate the following: −, negative reaction; ±, weak rection; +, positive reaction after 24, 48 and 72 h, respectively.

		Pentoses			Hexoses				6-Deoxy Hexoses		Sugar Alcohols						Disaccharides							Trisaccharides	
	Carbohydrate►	ARA	RIB	XYL	GAL	GLU	FRU	MNE	FUC	RHA	XLT	MAN	SOR	NAG	AMY	SAL	CEL	MAL	LAC	MEL	SAC	TRE	INU	MLZ	RAF
Ref.	LMG9476	−−−	−−−	−−−	+++	+++	+++	+++	−−−	−−−	−−−	+++	+++	+++	−−−	+++	−−−	+++	+++	+++	+++	+++	−−−	−−−	+++
	LMG9477	−−−	−−−	−−−	+++	+++	+++	+++	−−−	+++	−−−	+++	+++	+++	−−−	−−−	−−−	+++	+++	+++	+++	+++	−−−	−−−	+++
pigeons	P2a	−−−	−−−	−−−	+++	+++	+++	+++	−−−	+++	−−−	+++	−−−	+++	−−−	−−−	−−−	+++	+++	+++	+++	+++	−−−	−−−	+++
	P3a	−−−	−−−	−−−	+++	+++	+++	+++	−−−	+++	−−−	+++	−−−	+++	−−−	−−−	−−−	+++	+++	+++	+++	+++	+++	−−−	+++
	P 8a	−−−	−−−	−−−	+++	+++	+++	+++	−−−	±++	−−−	+++	−−−	±++	−−−	−−−	−−−	+++	+++	+++	+++	−−−	+++	−−−	+++
	P 16a	−−−	−−−	−−−	+++	+++	+++	+++	−−−	±++	−−−	+++	±++	+++	−−−	−−−	−−−	+++	+++	+++	+++	−−+	+++	−−−	+++
	P 18a	−−−	−−−	−−−	+++	+++	+++	+++	−−−	±++	−−−	+++	±++	±++	−−−	−−−	−−−	+++	+++	+++	+++	+++	−−−	−−−	+++
	P 21a	−−−	−−−	−−−	+++	+++	+++	+++	−−−	±++	−−−	±++	−−−	±++	−−−	−−−	−−−	+++	+++	+++	+++	+++	+++	−−−	+++
	P 23a	−−−	−−−	−−−	+++	+++	+++	+++	−−−	−−−	−−−	+++	+++	+++	−−−	−−−	−−−	+++	+++	+++	+++	+++	+++	−−−	+++
	Total [%]	0	0	0	100	100	100	100	0	86	0	100	43	100	0	0	0	100	100	100	100	86	71	0	100
chicken	Ch 4a	−−−	−−−	−−−	+++	+++	+++	+++	−−−	−−−	−−−	−++	−−−	−++	−−−	−−−	−−−	−−−	−++	−++	−++	−−−	−−−	−−−	−++
	Ch 8b	−−−	−−−	−−−	−++	+++	+++	+++	−−−	−−−	−−−	±++	−−−	+++	−−−	−−−	−−−	+++	+++	+++	+++	−−−	−−−	−−−	+++
	Ch 9b	−−−	−−−	−−−	+++	+++	+++	+++	−−−	−−−	−−−	+++	−−−	+++	−−−	−−−	−−−	+++	+++	+++	+++	+++	−−−	−−−	+++
	Ch 10a	−−−	−−−	−−−	+++	+++	+++	+++	−−−	−−−	−−−	+++	+++	+++	−−−	−−−	−−−	+++	+++	+++	+++	−−−	−−−	−−−	+++
	Ch10d	−−−	−−−	−−−	+++	+++	+++	+++	−−−	−−−	−−−	+++	+++	+++	−−−	−−−	−−−	+++	+++	+++	+++	−−−	−−−	−−−	+++
	Ch 24b	−−−	−−−	−−−	+++	+++	+++	+++	−−−	−−−	−−−	+++	−−±	+++	−−−	−−−	−−−	+++	+++	+++	+++	+++	−−−	−−−	+++
	Ch 37b	−−−	−−−	−−−	+++	+++	+++	+++	−−−	−−−	−−−	+++	+++	+++	−−−	−−−	−−−	+++	+++	+++	+++	+++	−−−	−−−	+++
	Ch 40a	−−−	−−−	−−−	+++	+++	+++	+++	−−−	−−−	−−−	+++	+++	+++	−−−	−−−	−−−	+++	+++	+++	+++	+++	−−−	−−−	+++
	Ch 50b	−−−	−−−	−−−	+++	+++	+++	+++	−−−	±++	−−−	+++	−−−	+++	−−−	+++	−−−	+++	+++	+++	+++	+++	−−−	−−−	+++
	Ch 50d	−−−	−−−	−−−	+++	+++	+++	+++	−−−	−−−	−−−	+++	−−±	+++	−−−	−−−	−−−	+++	+++	+++	+++	+++	−−−	−−−	+++
	Total: [%]	0	0	0	100	100	100	100	0	10	0	100	50	100	0	10	0	90	100	100	100	60	0	0	100
turkey	T 2a	−−−	−−−	−−−	+++	+++	+++	+++	−−−	−−−	−−−	+++	−−±	+++	−−−	−−−	−−−	+++	+++	+++	+++	+++	−−−	−−−	+++
	T 3a	−−−	−−−	−−−	+++	+++	+++	+++	−−−	−−−	−−−	−++	−−−	−++	−−−	−−−	−−−	−++	−++	−++	−++	−−−	−−−	−−−	−++
	T 7c	−−−	−−−	−−−	+++	+++	+++	+++	−−−	−−−	−−−	+++	−−−	−−−	−−−	−−−	−−−	+++	+++	+++	+++	−−−	−−−	−−−	+++
	T 17f	−−−	−−−	−−−	+++	+++	+++	+++	−−−	−−−	−−−	+++	−−−	−−−	−−−	−−−	−−−	+++	+++	+++	+++	−−−	−−−	−−−	+++
	T 18a	−−−	−−−	−−−	+++	+++	+++	+++	−−−	−−−	−−−	+++	+++	−−−	−−−	−−−	−−−	+++	+++	+++	+++	−−−	−−−	−−−	+++
	T 21a	−−−	−−−	−−−	+++	+++	+++	+++	−−−	−−−	−−−	−−±	−−−	−−±	−−−	−−−	−−−	+++	±±+	+++	+++	−−−	−−−	−−−	+++
	T 22a	−−−	−−−	−−−	+++	+++	+++	+++	−−−	−−−	−−−	+++	−−−	−−−	−−−	−−−	−−−	±++	−++	−++	+++	−−−	−−−	−−−	−−−
	T 27	−−−	−−−	−−−	+++	+++	+++	+++	−−−	−−−	−−−	+++	−−−	+++	−−−	−−−	−−−	+++	+++	+++	+++	−−−	−−−	−−−	+++
	T 31a	−−−	−−−	−−−	+++	+++	+++	+++	−−−	−−−	−−−	+++	+++	−−−	−−−	−−−	−−−	−−±	+++	+++	+++	−−−	−−−	−−−	+++
	Total: [%]	0	0	0	100	100	100	100	0	0	0	89	22	33	0	0	0	89	100	100	100	11	0	0	89
gees	G 2K	−−−	−−−	−−−	+++	+++	+++	+++	−−−	−−−	−−−	+++	+++	+++	+++	+++	+++	+++	−−±	−++	+++	+++	−−−	+++	−−−
	G 19a	−−−	−−−	−−−	+++	+++	+++	+++	−−−	+++	−−−	+++	−−±	+++	−−−	−−−	−−−	+++	+++	±++	+++	+++	−−−	−−−	+++
	G 24a	−−−	−−−	−−−	+++	+++	+++	+++	−−−	−−−	−−−	+++	+++	+++	−−±	−−−	−−−	+++	+++	+++	+++	+++	−−−	−−−	+++
	G 24b	−−−	−−−	−−−	+++	+++	+++	+++	−−−	−−−	−−−	+++	+++	+++	−−+	+++	−−−	+++	+++	+++	+++	+++	−−−	−−−	+++
	G 31a	−−−	−−−	−−−	+++	+++	+++	+++	−−−	−−−	−−−	+++	+++	+++	−−−	−−−	−−−	+++	+++	+++	+++	+++	−−−	−−−	+++
	G 39a	−−−	−−−	−−−	+++	+++	+++	+++	−−−	−−−	−−−	+++	+++	+++	−−−	−−−	−−−	+++	+++	+++	+++	+++	−−−	−−−	+++
	G 50b	−−−	−−−	−−−	+++	+++	+++	+++	−−−	−−−	−−−	+++	+++	+++	−−−	+++	−−−	+++	+++	+++	+++	+++	−−−	−−−	+++
	Total: [%]	0	0	0	100	100	100	100	0	14	0	100	86	100	14	43	14	100	86	100	100	100	0	14	86
	Total: [%]	0	0	0	100	100	100	100	0	24	0	97	48	82	3	12	3	94	97	100	100	61	15	3	94

**Table A3:** Analysis of DNA sequence and putative protein sequences of Abp118 bacteriocin.

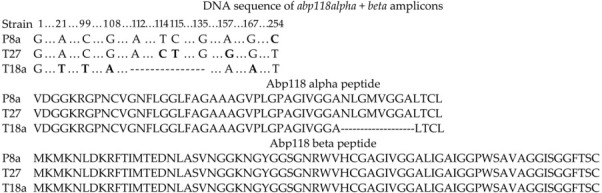

**Table A4:** The ability of avian *L. salivarius* strains to grow on MRS broth supplemented with 1% or 2% ox gall after 24 h and 48 h.

Isolate	Host Source	1% Bile	1% Bile	2% Bile	2% Bile
		24 h	48 h	24 h	48 h
LMG 9476	human	<3%	5%	<3%	6%
LMG 9477	human	<3%	5%	<3%	4%
P2a	pigeon	9%	9%	9%	11%
P3a	pigeon	8%	9%	8%	9%
P8a	pigeon	4%	8%	<3%	7%
P16a	pigeon	7%	8%	4%	12%
P18a	pigeon	9%	7%	8%	8%
P21a	pigeon	<3%	6%	<3%	6%
P23a	pigeon	6%	6%	6%	6%
Ch4a	chicken	4%	7%	<3%	5%
Ch8b	chicken	<3%	<3%	<3%	<3%
Ch9b	chicken	<3%	<3%	<3%	<3%
Ch10a	chicken	<3%	<3%	<3%	<3%
Ch10d	chicken	<3%	<3%	<3%	<3%
Ch24b	chicken	<3%	<3%	<3%	<3%
Ch37b	chicken	4.5%	13%	3.5%	12%
Ch40a	chicken	26%	38%	24%	37%
Ch50b	chicken	<3%	10%	5%	10%
Ch50d	chicken	8%	6%	5%	4.5%
T2a	turkey	5%	4.5%	4%	4%
T3a	turkey	14%	6%	8%	6%
T7c	turkey	<3%	<3%	<3%	<3%
T17f	turkey	<3%	4%	<3%	<3%
T18a	turkey	<3%	<3%	<3%	<3%
T21a	turkey	<3%	<3%	<3%	<3%
T22a	turkey	<3%	<3%	<3%	<3%
T27	turkey	<3%	10%	<3%	3.5%
T31a	turkey	5.5%	10%	<3%	6%
G2K	goose	100%	100%	100%	100%
G19a	goose	13%	13%	13%	17%
G24a	goose	13%	15%	13%	16%
G24b	goose	11%	11%	9%	10%
G31a	goose	14.5%	17%	13%	17%
G39a	goose	4.5%	6%	<3%	7%
G 50b	goose	8%	11%	7%	13%
